# Epidemiological and Genomic Characterisation of Middelburg and Sindbis Alphaviruses Identified in Horses with Febrile and Neurological Infections, South Africa (2014–2018)

**DOI:** 10.3390/v14092013

**Published:** 2022-09-11

**Authors:** Isabel Fourie, Jumari Snyman, June Williams, Arshad Ismail, Petrus Jansen van Vuren, Marietjie Venter

**Affiliations:** 1 Department of Medical Virology, University of Pretoria, Pretoria 0031, South Africa; 2 Department of Paraclinical Sciences, University of Pretoria, Pretoria 0110, South Africa; 3 Sequencing Core Facility, National Institute of Communicable Diseases (NICD), Johannesburg 2192, South Africa; 4 Department of Biochemistry and Microbiology, University of Venda, Thohoyandou 0950, South Africa; 5 Australian Centre for Disease Preparedness, CSIRO-Health and Biosecurity, Geelong 3220, Australia

**Keywords:** Alphavirus, Middelburg virus, Sindbis virus, horse, zoonotic, arbovirus, genome

## Abstract

Although Old World alphaviruses, Middelburg- (MIDV) and Sindbis virus (SINV), have previously been detected in horses and wildlife with neurologic disease in South Africa, the pathogenesis and clinical presentation of MIDV and SINV infections in animals are not well documented. Clinical samples from horses across South Africa with acute or fatal neurologic and febrile infections submitted between 2014–2018 were investigated. In total, 69/1084 (6.36%) and 11/1084 (1.01%) horses tested positive for MIDV and SINV, respectively, by real-time reverse transcription (RT) PCR. Main signs/outcomes for MIDV (*n* = 69): 73.91% neurological, 75.36% fever, 28.99% icterus and anorexia, respectively, 8.70% fatalities; SINV (*n* = 11): 54.54% neurological, 72.73% fever, 36.36% anorexia and 18.18% fatalities. MIDV cases peaked in the late summer/autumn across most South African provinces while SINV cases did not show a clear seasonality and were detected in fewer South African provinces. MIDV could still be detected in blood samples via RT-PCR for up to 71,417 and 21 days after onset of signs in 4 horses respectively, suggesting prolonged replication relative to SINV which could only be detected in the initial sample. Phylogenetic analyses based on partial sequences of the nsP4 (MIDV *n* = 59 and SINV *n* = 7) and E1 (MIDV *n* = 45) genes, as well as full genome sequences (MIDV *n* = 6), clustered the MIDV and SINV strains from the present study with previously detected strains. MIDV infection appears to be more prevalent in horses than SINV infection based on RT-PCR results, however, prevalence estimates might be different when also considering serological surveillance data.

## 1. Introduction

The Alphavirus genus (Togaviridae family) members are broadly divided into New World viruses that have traditionally been associated with neurological disease, and Old World members that are traditionally associated with fever and arthralgia [1,2].

Sindbis virus (SINV) was first isolated from *Culex univitattus* mosquitoes in 1952 and members of the *Culex* genus were subsequently shown to be the primary vectors and birds the amplifying hosts [3,4,5]. Although SINV is widespread across Africa, Eurasia and Australia, most human disease is reported from South Africa and Northern Europe [4]. One of the largest human outbreaks in South Africa occurred during 1974 in the Karoo and Northern Cape, resulting in thousands of human infections which co-circulated with West Nile virus [6]. Smaller clusters of SINV infections have since been detected across South Africa with annual cases detected in the highveld [7,8]. Symptoms usually include rash, fever, arthralgia, myalgia and fatigue with musculoskeletal symptoms, often persisting for prolonged periods [4]. 

Middelburg virus (MIDV) was first isolated from *Aedes (Ae) caballus* and *Ae. Banksinella* sp. mosquitoes in 1957 in Middelburg, Eastern Cape Province of South Africa, during an outbreak of febrile illness and fatalities in sheep [9]. Although the outbreak was determined to be caused by Wesselsbron virus (Flavivirus), inoculation of MIDV into sheep resulted in viremia [9]. MIDV was later detected in a horse during an outbreak of fever and icterus in South Africa in 1974 [10,11] and in 2007 MIDV was isolated from the spleen of a horse that died in Zimbabwe in 1993 [12]. The Zimbabwean horse presented with signs similar to those caused by African horse sickness virus (AHSV, family Reoviridae), which included increased body temperature, tachycardia, pulmonary signs and general edema, especially severe in the head and neck [12]. 

SINV and MIDV infections have been associated with severe clinical signs in animals in South Africa and were identified in 52 of 623 horses with febrile or neurologic disease between 2008- 2013 [13]. SINV infection was detected in 8/623 (1.28%) horses with fever, ataxia, recumbency, muscle weakness, depression and listlessness as well as fatalities, although most (2/3) fatal cases were associated with co-infections with West Nile virus (WNV) [13]. MIDV infection was detected in 44/623 (7.06%) horses with acute febrile (13/44; 29.54%) and/or neurologic manifestations (30/44; 68.18%) and fatalities (12/44; 27.27%) where 2/12 fatalities were associated with WNV and AHSV co-infections, respectively. MIDV was detected in the brain of all MIDV positive horses with available central nervous system (CNS) tissue. Signs in less severe cases included fever, stiffness, swollen limbs and depression [13]. 

Another South African study reported SINV and MIDV infections in wildlife and non-equine domestic animals with unsolved neurologic, febrile, and respiratory signs or sudden unexpected death [14]. Between 2010–2018, SINV was detected in *n* = 9/608 (1.48%) of these animals including wildlife (buffalo, sable antelopes, rhinoceroses, giraffes, European wild boar, blesboks and genets) and non-equine domestic animals (porcine and ovine). All SINV positive samples were detected in postmortem tissue. Neurological signs were reported in all clinically ill animals and included ataxia, paralysis, tongue paralysis and recumbency while other signs/outcomes for SINV infection included fever, dyspnoea, sudden unexpected death and abortion [14]. MIDV was detected in *n* = 23/608 (3.78%) of these animals including white rhinoceros, buffalo, domestic bovids, warthogs, lions, birds (lemon dove and blue crane), sable antelope, waterbuck and genets. Of the MIDV positive samples, *n* = 20/23 (86.95%) were detected in postmortem specimens. Neurological signs were reported for all MIDV infections and included ataxia, paralysis, quadriparesis and recumbency and other signs/outcomes were fever and sudden unexpected death [14].

This suggests that both MIDV and SINV have a wide host range and zoonotic potential. Although the amplifying host for SINV is known to be birds, the epidemiology of MIDV is not well understood, and the amplifying hosts are not known.

This study aimed to investigate the epidemiology including clinical presentation, geographic distribution, and seasonality of MIDV and SINV infections in horses presenting with febrile and neurological disease in South Africa over 5 years. The period for which virus could still be detected was determined retrospectively in 4 MIDV cases. The molecular epidemiology and genomic characteristics of isolates from mild and severe cases were determined using partial sequencing of the nsP4 and E1 genes and full genome sequencing of 6 MIDV samples.

## 2. Materials and Methods

### 2.1. Ethical Considerations

Ethical approval was obtained (31 August 2017) from the University of Pretoria’s animal research ethics committee, project number H013-17. The study was approved under Section 20 (no. 12/11/1/1) by the Department of Agriculture, Forestry and Fisheries (DAFF) (Currently known as Department of Agriculture Land Reform and Rural Development (DALRRD).

### 2.2. Specimens

Samples received between January 2014–December 2018 from horses displaying acute febrile/neurologic signs as well as fatalities related to these signs were routinely submitted by veterinarians or veterinary pathologists as part of a passive surveillance program to the Zoonotic Arbo- and Respiratory Virus program (ZARV program, Centre for Viral Zoonoses, Department of Medical Virology, University of Pretoria, South Africa). Samples were accompanied by a specimen submission form containing demographic and clinical information provided by a qualified veterinarian that tended to each case.

### 2.3. Viral RNA Extraction, Detection, and Sanger Sequencing

RNA was extracted from serum (clotted blood), plasma and whole blood (EDTA (Ethylenediaminetetraacetic acid) samples using the QIAmp viral RNA Kit (QIAGEN™, Hilden, Germany) and the RNeasy Plus Kit was used for extraction of tissue samples (QIAGEN™, Hilden, Germany) according to manufacturer’s instructions.

Primers/probes used in the current study are summarized in Table 1. Extracted RNA was subjected to screening using a published, semi-quantitative, nested real-time Alphavirus PCR which results in a 198-base pair (bp) fragment of the nsP4 gene [13,15]. Following detection of MIDV/SINV positives via the screening, PCR, MIDV and SINV specific nested primers [14] were used to increase the nsP4 region from 198 bp to 347 bp to improve phylogenetic analysis. For differential diagnosis all specimens were also screened for flaviviruses [16], Equine encephalosis virus (EEV) (EEV screened for either at the ZARV or Equine Research Centre, University of Pretoria) and Shuni virus [17].

A 550 bp fragment of the MIDV E1 gene was also amplified using a combination of newly designed and previously published [13] primers to investigate recombination events. The first round PCR was set up using SuperScript™ III One-Step RT-PCR System with Platinum™ Taq DNA polymerase (Thermo Fisher Scientific, Waltham, MA, USA) with 10 µL RNA, 2× Reaction Mix Superscript [1×] final, 20 µM primer [0.4 µM] final (MIDVEF and MIDV(ER), 2 µL SuperScript™ III RT/Platinum™ Taq Mix and ddH_2_O (double distilled water) up to a final volume of 50 µL. Cycling conditions: 50 °C for 30 min, 94°C for 2 min, (94 °C for 15 s, 52 °C for 30 s, 68 °C for 1 min) × 40 cycles, 68 °C for 5 min, 4 °C hold.

The nested MIDV E1 PCR was set up using 2 µL first round product, 10× DreamTaq Buffer (Thermo Fisher Scientific) [1×] final, 10 mM dNTP Mix [0.4 mM] final, 10 µM primer (MIDVEN 9F and MIDV10911 EN) [0.2 µM] final, DreamTaq Polymerase 500 U (Thermo Fisher Scientific, Waltham, MA, USA) [2.5 U] final and nuclease and RNase free ddH_2_O up to a final volume of 50 µL. Cycling conditions 95 °C for 2 min, (95 °C for 30 s, 52 °C for 30 s, 72 °C for 1 min) × 40 cycles, 72 °C for 7 min, 4 °C hold. PCR products were purified using the Zymoclean™ Gel DNA recovery kit (Zymo Research, Irvine, CA, USA) according to the manufacturer’s instructions and submitted for Sanger sequence analysis (Inqaba Biotechnology or University of Pretoria DNA Sanger sequencing facility, South Africa).

### 2.4. RNA Replication for MIDV and SINV Positive Follow-Up Specimens

Follow-up samples were received from 18 horses that tested positive for MIDV and 1 horse that tested positive for SINV with initial submission in 2017/2018. These follow-up bloods were used to determine the period for which MIDV and SINV can be detected using the nested real-time PCRs as described above.

### 2.5. Virus Isolation and Sequence-Independent Single-Primer Amplification (SISPA) with Rapid Amplification of cDNA Ends (RACE) for Full Genome Sequencing

All virus isolations were attempted in the BSL-3 facility at the University of Pretoria’s Centre for Viral Zoonosis. The isolation of MIDV and SINV from positive whole blood, serum or tissue samples was attempted by inoculation of samples on Vero cells [9,18].

Briefly, Vero cells were grown in 25 cm^2^ flasks maintained in Eagle’s Minimum Essential Medium (EMEM, Sigma Aldrich, Saint Louis, MO, USA), supplemented with 10% (*v*/*v*) Foetal Bovine Serum (FBS, Sigma Aldrich) and 1× (*v*/*v*) MycoZap Plus-CL (LONZA, Basel, Switzerland) at 37 °C, 5% CO_2_ until a monolayer of approximately 80% was obtained. Supernatant was discarded and cells washed 3 times with PBS (Phosphate Buffered Saline, Sigma Aldrich), 200 µL of whole blood, serum or homogenised tissue adsorbed for 1 h at 37 °C, 5% CO_2_ followed by addition of 5 mL EMEM, supplemented with 2% FBS and MycoZap Plus-CL. Cells were monitored for cytopathic effect (CPE) over 7 to 10 days. Subsequent passaging was performed by using 500 µL of supernatant from the previous passage, following 3 times freeze thawing at −80 °C and clarification by centrifugation, for inoculation on new cells.

Supernatant of cultures showing CPE was used for viral RNA extraction (RNeasy Plus Kit, QIAGEN™, Hilden, Germany) and positive isolates were confirmed by the nested real-time PCR as described above. Twelve ml of confirmed MIDV positives supernatants were inoculated on 75 cm^2^ tissue culture flasks in triplicate for preparation of stock virus and full genome sequencing. Approximately 36 mL of infected supernatant was filtered through a 0.45 µM bacterial filter (Sartorius Stedim Biotech, Göttingen, Germany). Supernatant filtrate was concentrated to 500 µL using Ultra-15 Centrifugal Units with 10 kDa molecular weight cut-off (Amicon, Merck, Darmstadt, Germany). RNA was extracted using a 1:3 ratio of concentrated supernatant to Trizol-LS reagent (Sigma-Aldrich, Saint Louis, MO, USA) in conjunction with the Direct-Zol RNA kit (Zymo Research, Irvine, CA, USA) including the in-column DNase treatment. This was followed by purification using the RNA Clean and Concentrator-5 kit (Zymo Research, Irvine, CA, USA) as per manufacturer’s instructions. RNA was converted to cDNA and amplified using the SISPA (RACE) technique [19]. SISPA is an unbiased next generation sequencing technique, but it has previously been reported that 3′ and 5′ ends of gene segments were missing using the SISPA technique, while a combination of SISPA and rapid amplification of cDNA ends (SISPA-RACE) resulted in full genomes. Thus, the SISPA-RACE technique was used as described previously [19].

For samples for which no culture isolates could be obtained, RNA was extracted directly from whole blood samples using Trizol-LS reagent and the Direct-Zol RNA kit as described above for culture isolates followed by SISPA-RACE amplification for attempted full genome sequencing.

### 2.6. Illumina Full Genome Sequencing

Full genome sequencing was conducted at the National Institute for Communicable Diseases (NICD) sequencing core facility in Sandringham, Johannesburg, South Africa. Generated cDNA was sent to the NICD where libraries were prepared using the Illumina Nextera DNA preparation kit followed by sequencing (2 × 300 bp) on a llumina Miseq instrument (Illumina, Inc., San Diego, CA, USA).

### 2.7. Phylogenetic Analysis

The larger nsP4 amplicon for MIDV and SINV (347 bp) and the E1 gene amplicon for MIDV (550 bp) strains obtained from PCR positive samples were used for phylogenetic analysis. Both Sanger sequenced fragments and full genome sequences were analysed using CLC main workbench v8 (https://www.qiagenbioinformatics.com/, accessed on 16 August 2019). For full genomes, failed reads and low-quality scores were discarded and reads mapped against reference sequence MIDV SAE25/11 (GenBank accession number KF680222.1). Full genome sequences were aligned within the structural and non-structural open reading frames (ORFs) as described previously [20]. Briefly, the two ORFs were obtained from full genome sequences for currently described MIDV strains and concatenated. Alphavirus sequences from the different antigenic complexes were downloaded from Genbank (Accession numbers Supplementary Table S1). The C terminal of the nsP3 and the N terminal of the capsid sequences were removed to allow for better phylogenetic analysis as described by previous authors since these regions contain extensive divergence hindering reliable alignments [20,21].

Multiple sequence alignments were performed using the online MAFFT v7 alignment tool available on https://mafft.cbrc.jp/alignment/software/ (accessed on 10 December 2020) [22]. Optimal evolutionary trees were estimated using jModelTest2 v2.1.6 [23] and Bayesian analysis conducted using BEAST v1.10.4 [24] accessed through the CIPRES Science Gateway [25] utilizing a GTR+G+I model and uncorrelated relaxed clock. Separate phylogenetic analysis was conducted for the nsP4 fragment, the E1 fragment and the full genome sequences of nsP’s (ORF1), sP’s (ORF2) and concatenated ORF1 and ORF2, respectively. Phylogenetic trees were visualized using TreeGraph2 [26]. Pairwise distance (p-distance) analysis was calculated in MEGA 7 (Molecular Evolutionary Genetics Analysis software) [27] and used to determine percentage identities between different MIDV and SINV strains. Sequences were submitted to GenBank. Accession numbers for presently described MIDV and SINV strains and reference sequences used are indicated in Supplementary Table S1.

### 2.8. Statistical Analysis

Data and statistical analyses were conducted in EpiInfo™ (version 7.2.0.1) using Fisher’s exact test with a 95% confidence interval (CI) and odds ratios (OR) to calculate the association between clinical signs and virus infection [28].

### 2.9. Recombination Analysis

The recombination analysis program RDP4 [29], which alongside RDP [30] also uses other recombination analysis programs including GENECOV [31], Chimaera [32], Sciscan [33], Bootscan [34], MaxChi [35] and 3Seq [36], was used to detect recombination events in the structural region of alphavirus sequences.

## 3. Results

### 3.1. MIDV and SINV Detection and Clinical Disease Description

Over a 5-year period from January 2014–December 2018, a total of 6.36% (69/1084) specimens meeting the case definition tested positive for MIDV and 1.01% (11/1084) for SINV. Sample types received included whole blood, serum, cerebrospinal fluid (CSF), post-mortem central nervous system tissue (CNS) (brain and spinal cord), respiratory organs (lungs) and visceral organs (spleen and liver). MIDV (*n* = 69) was detected in whole blood (61/69), serum (5/69), brain (1/69), spinal cord (1/69) and brain and placenta, respectively, of an aborted foetus (1/69). MIDV/WNV co-infections were detected in the brain and spinal cord samples (1/69 and 1/69, respectively) while only MIDV was detected in the brain and placenta of the aborted foetus. All SINV positives (*n* = 11) were detected in whole blood samples. Total co-infections were as follows: two MIDV/SINV (2/2 detected in whole blood), 6 MIDV/WNV (4/6 detected in whole blood; 1/6 detected in brain; 1/6 detected in spinal cord), 4 MIDV/EEV (2/4 detected in whole blood; 2/4 detected in serum), 4 MIDV/AHSV (4/4 detected in whole blood) and 1 SINV/AHSV/EEV (1/1 detected in whole blood). Fatal cases are indicated in Table 2.

Clinical signs observed for MIDV (*n* = 69) and SINV (*n* = 11) positives are indicated in Table 3 with statistically significant (*p* < 0.05) signs indicated in bold. Of the MIDV positives 8.70% (6/69) were fatal and death was significantly associated with MIDV infection (*p* = 0.004) although 66.66% (4/6) of the fatalities were MIDV/WNV co-infections and the remaining 33.33% (2/6) were MIDV single infections

Neurological signs were present in 73.91% (*p* = 0.188; OR 1.48; 95% CI 0.85–2.59) of MIDV positives with ataxia as the most frequently observed (49.23%; *p* = 0.074; OR 1.56; 95% CI 0.96–2.55) neurological sign. Stiffness was the only neurological sign to show a statistically significant association with MIDV infection (8.70%; *p* = 0.00001; OR 13.71; 95% CI 4.47–42.02).

The majority of MIDV positive horses also presented with fever (75.36%; *p* = 0.00025; OR 2.81; 95% CI 1.60–4.92) with ‘fever and neurological signs’ (55.07%; *p* = 2 × 10^−10^; OR 5.25; 95% CI 3.18–8.66), being significantly associated with MIDV infections. Other statistically significant signs included: icterus (28.99%; *p* = 0.00021; OR 3.1; 95% CI 1.75–5.40), anorexia/inappetence (28.99%; *p* = 0.039; OR 1.78; 95% CI 1.03–3.07) and pallor (14.49%; *p* = 0.010; OR 2.69; 95% CI 1.31–5.53). For SINV 18.18% (2/11) resulted in fatalities of which one was a SINV/AHSV/EEV co-infection and the other a single SINV infection. Neurological signs were present in 54.54% (*p* = 0.522; OR 0.42; 95% CI 0.12–1.38) of SINV positives (*n* = 11), with ataxia 27.27% (*p* = 0.543; OR 0.58; 95% CI 0.15–2.21) followed by recumbency 18.18% (*p* = 0.678; OR 1.24; 95% CI 0.26–5.79), being the most frequently observed neurological signs. Facial nerve paralysis (9.09%, *p* = 0.040 OR 35.67; 95% CI 3.41–372.98) and nasal discharge (18.18%; *p* = 0.021; OR 12.26; 95% CI 2.48–60.64) were significantly associated with SINV infection. Fever was present in 72.73% (*p* = 0.237; OR 2.33; 95% CI 061–8.81) of SINV infections.

Follow-up specimens were received during 2017 and 2018 for *n* = 18/49 MIDV (2017:42 + 2018:7 MIDV positives) and *n* = 1/1 SINV (2017: 0 SINV positives + 2018:1 SINV positive). Of these follow-up specimens, 4/18 still tested positive for MIDV on day 7, 14 and 17 and 21, respectively, after initial onset of signs in follow-up specimens during 2017/2018 and none for SINV using the real-time screening RT-PCR described above. Of the 4 MIDV positive follow-up samples, one horse still presented with fever 4 days after the initial specimen was sampled and another still had icterus 7 days after the initial specimen was sampled. The SINV follow-up specimen was received 14 days after initial onset of signs and was SINV negative via RT-PCR at this point. Table 4 summarizes clinical information of the four MIDV positive follow-up specimens for initial and follow-up samples.

### 3.2. Seasonality of Alphaviruses in South Africa

**Figure 1:**
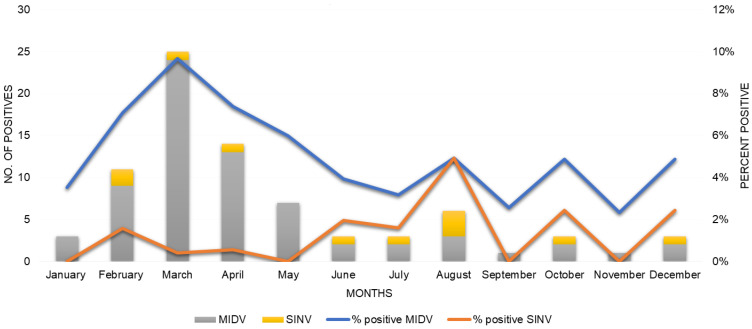
Seasonal detection of Middelburg virus (MIDV) (*n* = 69) and Sindbis virus (SINV) (*n* = 11) positive infections in horses from South Africa between January 2014–December 2018. Number of MIDV positives indicated by grey bars and percent positivity indicated by blue line. Number of SINV positives indicated by yellow bars and percent positivity indicated by orange line.

MIDV infection was detected yearly from 2014–2018 with most MIDV positive cases detected in 2017 (9.84%, 42/427) followed by 2018 (5.15%; 7/136), 2015 (5.08%; 10/197), 2014 (4.62%; 9/195) and only one case detected in 2016 (0.78%, 1/129). For SINV cases the majority were detected in 2015 (4.06%; 8/197) followed by 2014 (1.03%; 2/195) and 2018 (0.76%,1/136) with no SINV cases detected during 2016 and 2017. As seen in Figure 1, MIDV was detected year-round with an increase in the number of cases seen in the late summer till early autumn months from February to May following the rainy season, with the highest positivity seen in March (9.67%, 24/248) and April (7.38%; 13/176). Since only 11 SINV cases were detected between 2014–2018 seasonality is more difficult to determine, but from the available data SINV was detected year-round except in January, May, September, and November with the highest percent positivity seen in August (4.91%, 3/61).

### 3.3. Geographic Distribution

**Figure 2:**
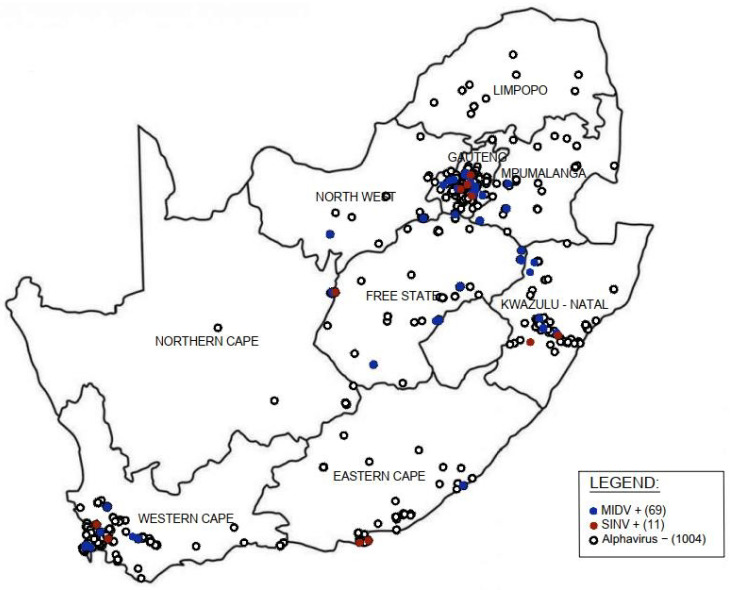
Map of Middelburg (MIDV)- and Sindbis virus (SINV) PCR positive infections reported in horses tested in South Africa from January 2014–December 2018. +: positive; −: negative.

Samples were received from all nine provinces within South Africa with MIDV infection detected in all provinces except Limpopo. The highest percent positives for MIDV were detected from the Free State (16.66%; 8/48), Mpumalanga (11.76%; 4/34), KwaZulu Natal (9.23%; 12/130), North West (8.10%; 3/37) and Gauteng (7.07%, 30/424). SINV positives were detected from Eastern Cape (3.12%; 2/64), Northern Cape (2.00%; 1/50), KwaZulu Natal (1.53%; 2/130), Gauteng (0.94%; 4/424) and Western Cape (0.83%; 2/239) provinces only (Figure 2).

### 3.4. Detection and Phylogenetic Analysis of Partial Sequences

**Figure 3:**
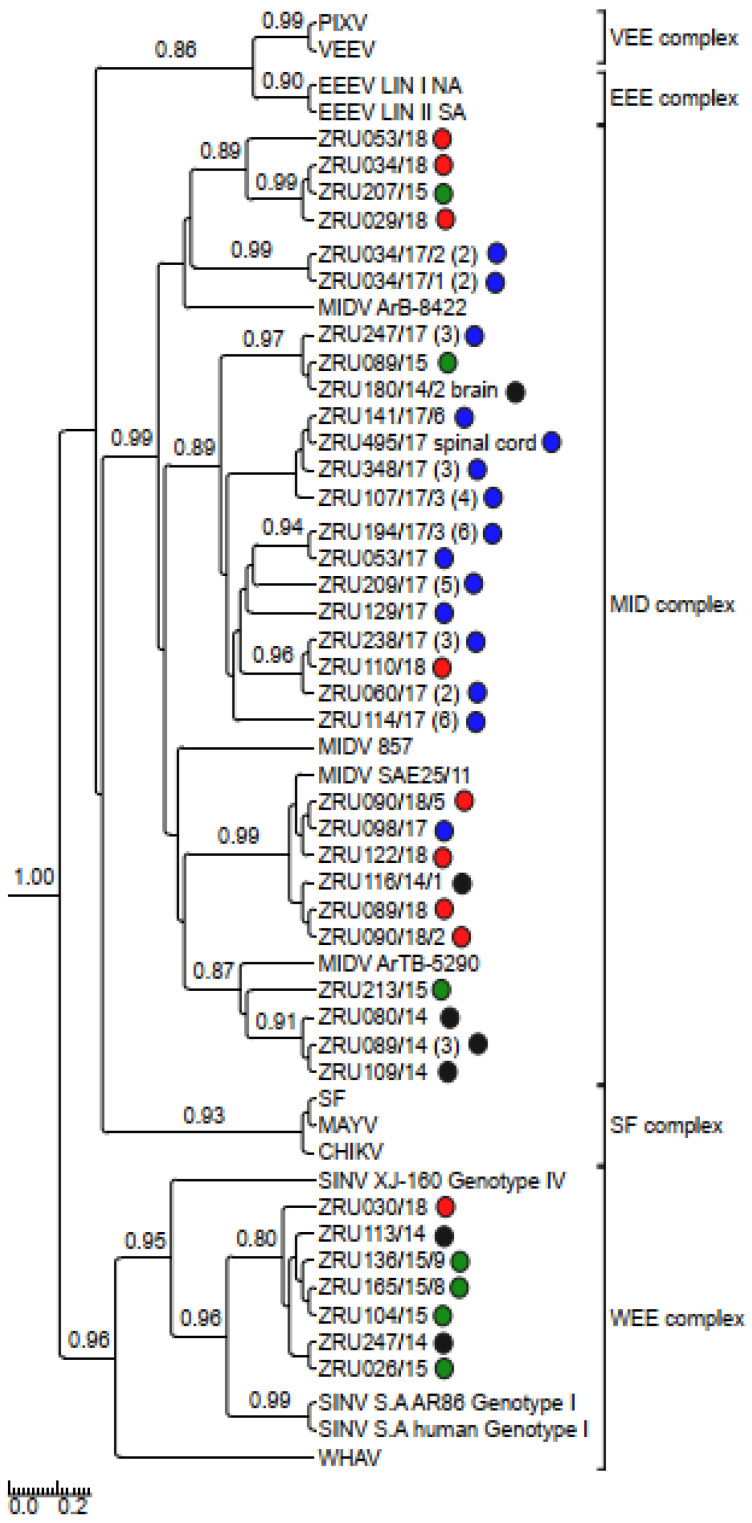
Rooted phylogenetic tree produced from Bayesian analysis using BEAST based on a 347-base pair nsP4 fragment (83 taxa, model GTR+G+I) of alphaviruses is shown. Posterior probabilities >0.7 are shown on major branches. Middelburg- and Sindbis virus positives described in current study indicated with ZRU numbers and circle colours correspond to year as follows: 2014 black, 2015 green, 2017 blue and 2018 red. All sequences obtained from whole blood or serum unless otherwise indicated. Number of sequences within the same clade for samples from the same year indicated in brackets. Alphavirus complexes indicated by brackets; tree is drawn to scale representing the number of nucleotide substitutions per site. CHIKV: Chikungunya virus; EEEV: Eastern equine encephalitis virus; MAYV: Mayaro virus; MIDV, Middelburg virus; PIXV: Pixuna virus; SFV, Semliki Forest virus; SINV: Sindbis virus; VEEV: Venezuelan equine encephalitis virus; WHAV: Whataroa virus; WEE: Western equine encephalitis.

**Figure 4:**
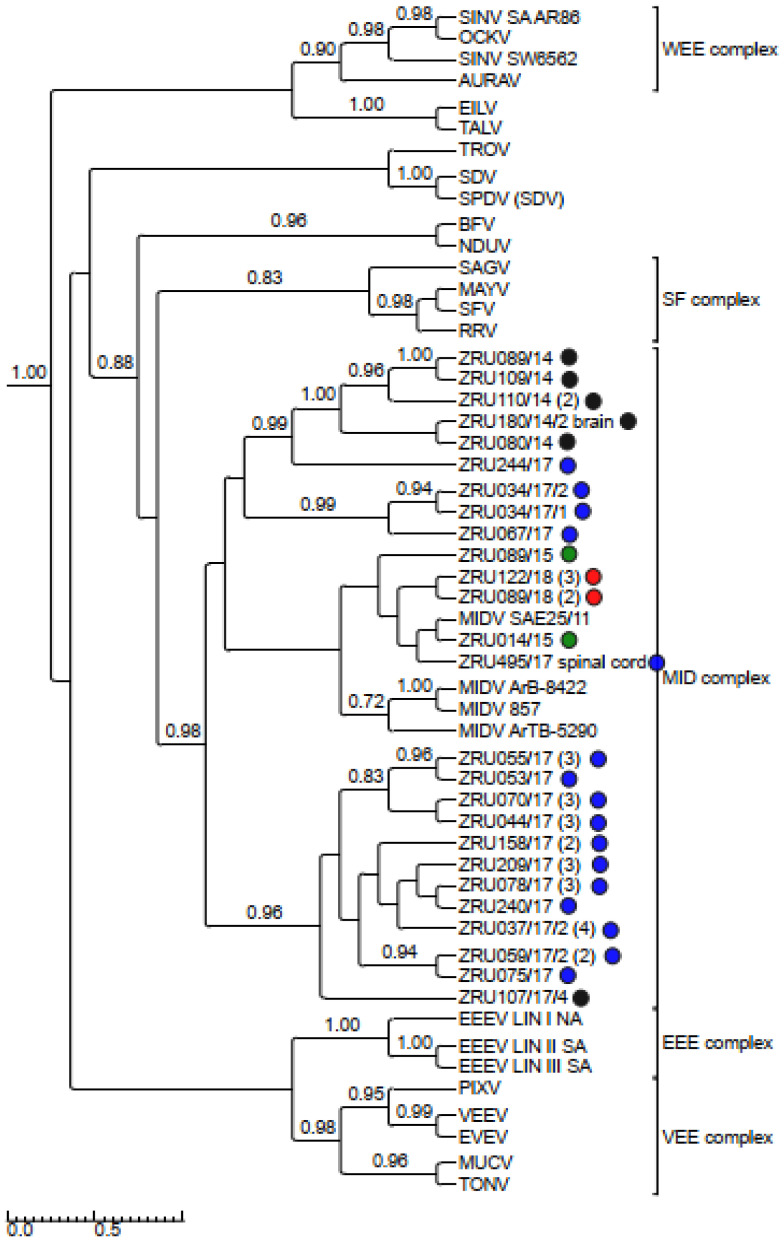
Rooted phylogenetic tree produced from Bayesian analysis using BEAST based on a 550-base pair E1 fragment (74 taxa, model GTR+G+I) of alphaviruses is shown. Posterior probabilities >0.7 are shown on major branches. Middelburg virus positives described in the current study are indicated with ZRU numbers and circle colours correspond to year as follows: 2014 black, 2015 green, 2017 blue and 2018 red. All sequences obtained from whole blood or serum unless otherwise indicated. Number of sequences within the same clade for samples from the same year indicated in brackets. Alphavirus complexes are indicated by brackets. The tree is drawn to scale representing the number of nucleotide substitutions per site. AURAV: Aura virus; BF: Barmah Forest virus; CHIKV: Chikungunya virus; EILV: Eilat virus; EEEV: Eastern equine encephalitis virus; EVEV: Everglades virus; MAYV: Mayaro virus; MIDV, Middelburg virus; MUCV: Mucambo virus; NDUV: Ndumu virus; OCKV: Ockelbo virus; PIXV: Pixuna virus; RRV: Ross river virus; SAGV: Sagiyama virus; SDV: Sleeping disease virus; SEV: Southern elephant seal virus; SFV, Semliki Forest virus; SINV: Sindbis virus; TALV: Taï Forest alphavirus; TONV: Tonate virus; TROV: Trocara virus; VEEV: Venezuelan equine encephalitis virus; WHAV: Whataroa virus; WEE: Western equine encephalitis.

MIDV/SINV sequences for either the smaller (198 bp) or larger (347 bp) nsP4 fragment were used to confirm all real time RT-PCR positives cases. Only sequences >200 bp (thus excluding the 198 bp nsP4 fragment) were submitted to GenBank (Supplementary Table S1). The 347 bp nsP4 (MIDV *n* = 59; SINV *n* = 7) fragment (Figure 3) as well as the 550 bp E1 fragment for MIDV (*n* = 45) (Figure 4) were used for phylogenetic analysis. Analysis of the nsP4 and E1 regions placed MIDV in its own complex with previously identified MIDV strains. Analysis of the E1 region showed MIDV strains from the same year mostly clustering together while that of the nsP4 region did not show a clear distinction between year and clusters. SINV identified in the current study formed a distinct cluster, showing the greatest similarity to Genotype I SINV strains, within the WEE complex. P-distance analysis percentage identities range from 94.2–100% for MIDV and 90.8–100% for SINV, respectively, comparing different strains in the nsP4 region and 95.6–100% for the MIDV E1 region.

### 3.5. Virus Isolations for Full Genome Sequence Analysis

Virus isolations were successful in Vero cells for 20/69 MIDV positive horses as confirmed by the presence of ≥70% CPE followed by detection via real-time RT-PCR as previously described. Four MIDV positive isolates and an additional two MIDV positive whole blood samples were selected for full genome sequence analysis based on amplification following first round PCR (vs detection only following nested PCR), severity of signs and location. CPE was generally visible by day 4 to 5 and all four isolates were passaged five times prior to virus isolations described in Section 2.5. There was no clear association between ability to isolate virus and severity of clinical signs. Virus isolation attempts were unsuccessful for SINV.

**Figure 5:**
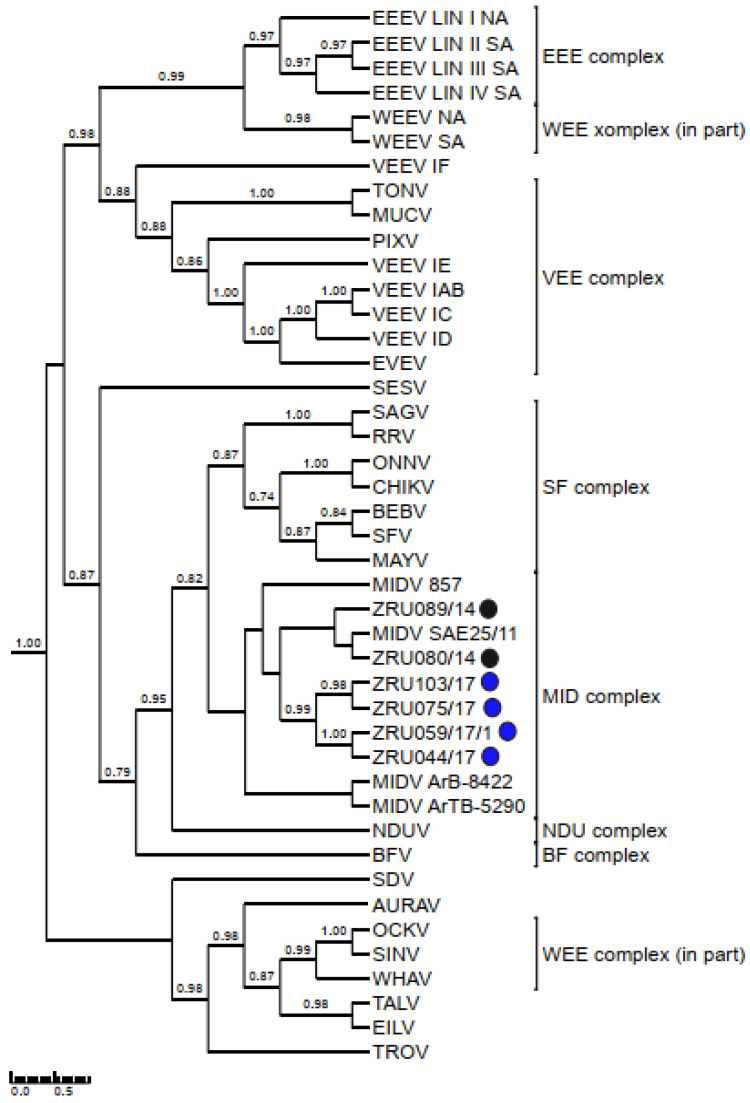
Rooted phylogenetic tree produced from Bayesian analysis using BEAST based on non-structural proteins (7236 base pairs) (33 taxa, model GTR+G+I) of alphaviruses is shown. Posterior probabilities >0.7 are shown on major branches. Sequences of horse Middelburg virus positives described in the current study are indicated with ZRU numbers and circle colours correspond to year sample was screened as follows: 2014 indicated in black (sequences obtained from whole blood samples) and 2017 (sequences obtained from culture isolates) indicated in blue. Alphavirus complexes are indicated by brackets. The tree is drawn to scale representing the number of nucleotide substitutions per site. AURAV: Aura virus; BF: Barmah Forest virus; CHIKV: Chikungunya virus; EILV: Eilat virus; EEEV: Eastern equine encephalitis virus; EVEV: Everglades virus; GETV: Getah virus; MAYV: Mayaro virus; MIDV, Middelburg virus; MUCV: Mucambo virus; NDUV: Ndumu virus; OCKV: Ockelbo virus; PIXV: Pixuna virus; RRV: Ross river virus; SDV: Sleeping disease virus; SEV: Southern elephant seal virus; SFV, Semliki Forest virus; SINV: Sindbis virus; TALV: Taï Forest alphavirus; TONV: Tonate virus; TROV: Trocara virus; VEEV: Venezuelan equine encephalitis virus; WHAV: Whataroa virus; WEE: Western equine encephalitis.

**Figure 6:**
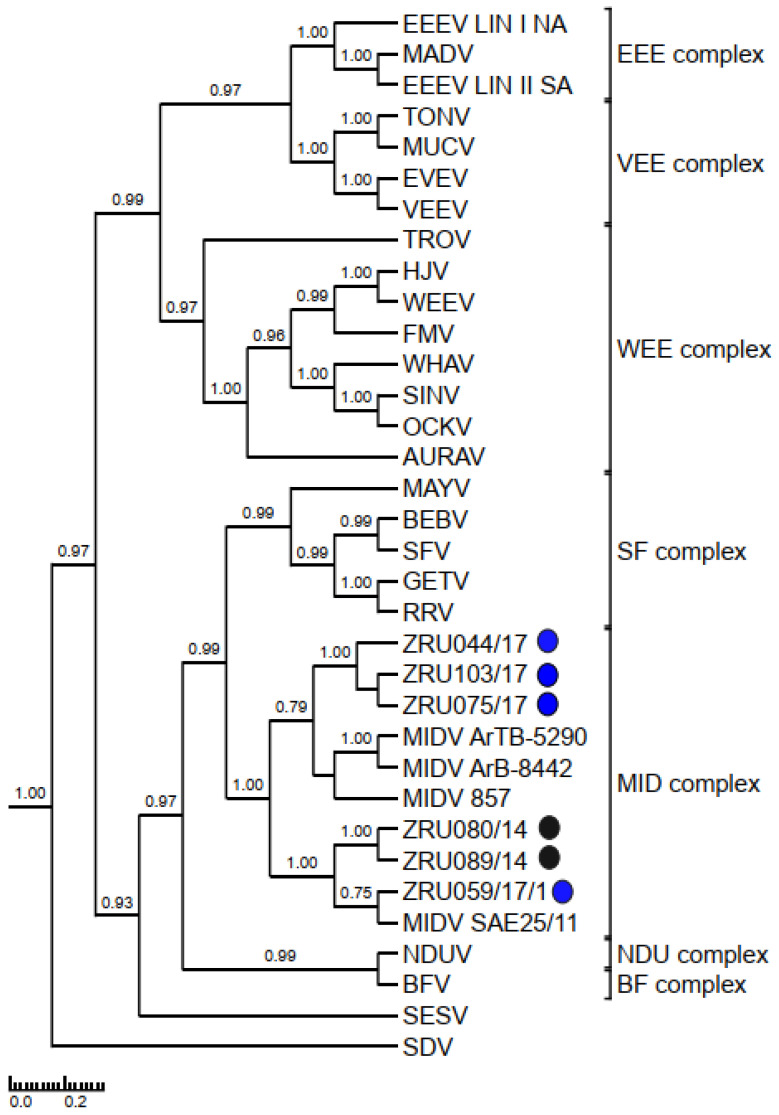
Rooted phylogenetic tree produced from Bayesian analysis using BEAST based on structural proteins (3777 base pairs) (35 taxa, model GTR+G+I) of alphaviruses is shown Posterior probabilities >0.7 are shown on major branches. Sequences of horse Middelburg virus positives described in the current study are indicated with ZRU numbers and circle colours correspond to year sample was screened as follows: 2014 indicated in black (sequences obtained from whole blood samples) and 2017 indicated in blue (sequences obtained from culture isolates). AURAV: Aura virus; BF: Barmah Forest virus; CHIKV: Chikungunya virus; EILV: Eilat virus; EEEV: Eastern equine encephalitis virus; EVEV: Everglades virus; GETV: Getah virus; MAYV: Mayaro virus; MIDV, Middelburg virus; MUCV: Mucambo virus; NDUV: Ndumu virus; OCKV: Ockelbo virus; PIXV: Pixuna virus; RRV: Ross river virus; SDV: Sleeping disease virus; SEV: Southern elephant seal virus; SFV, Semliki Forest virus; SINV: Sindbis virus; TALV: Taï Forest alphavirus; TONV: Tonate virus; TROV: Trocara virus; VEEV: Venezuelan equine encephalitis virus; WHAV: Whataroa virus; WEE: Western equine encephalitis.

**Figure 7:**
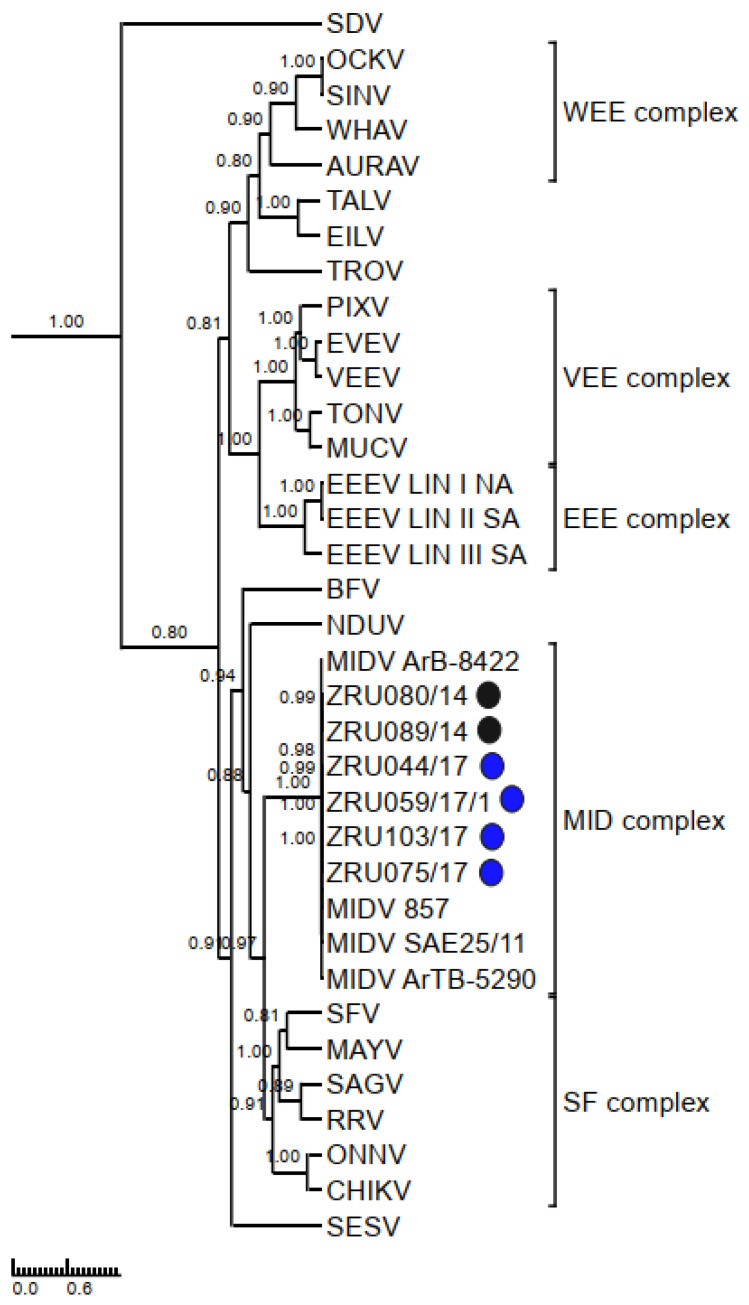
Rooted phylogenetic tree produced from Bayesian analysis using BEAST based on concatenated nucleotide sequences of non-structural (7236 base pairs) and structural protein (3777 base pairs) open reading frames (35 taxa, model GTR+G+I) of alphaviruses is shown. Probabilities >0.7 are shown on major branches. Sequences of horse Middelburg virus positives described in the current study are indicated with ZRU numbers and circle colours correspond to year sample was screened as follows: 2014 indicated in black (sequences obtained from whole blood samples) and 2017 indicated in blue (sequences obtained from culture isolates). Alphavirus complexes are indicated by brackets. The tree is drawn to scale representing the number of nucleotide substitutions per site. AURAV: Aura virus; BF: Barmah Forest virus; CHIKV: Chikungunya virus; EILV: Eilat virus; EEEV: Eastern equine encephalitis virus; EVEV: Everglades virus; GETV: Getah virus; MAYV: Mayaro virus; MIDV, Middelburg virus; MUCV: Mucambo virus; NDUV: Ndumu virus; OCKV: Ockelbo virus; PIXV: Pixuna virus; RRV: Ross river virus; SDV: Sleeping disease virus; SEV: Southern elephant seal virus; SFV, Semliki Forest virus; SINV: Sindbis virus; TALV: Taï Forest alphavirus; TONV: Tonate virus; TROV: Trocara virus; VEEV: Venezuelan equine encephalitis virus; WHAV: Whataroa virus; WEE: Western equine encephalitis.

**Figure 8:**
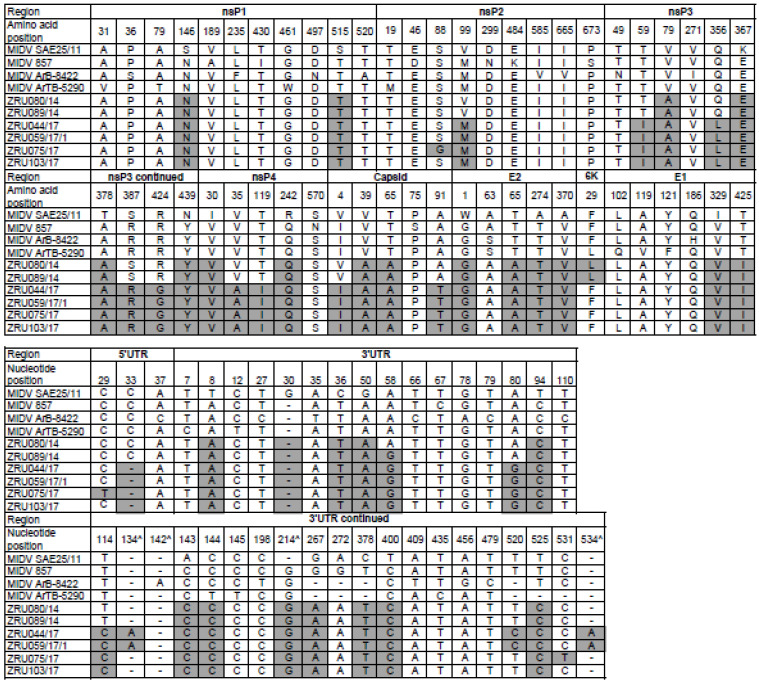
Amino acid and nucleotide sequence comparisons of the structural and non-structural proteins and UTR’s, respectively, between different Middelburg virus (MIDV) strains. Numbering refers to sequence positions of isolate SAE25/11. Changes in amino acid of ZRU080/14, ZRU089/14, ZRU044/17, ZRU059/17/1, ZRU075/17, and ZRU103/17 as compared to MIDV SAE25/11 are highlighted in grey. Deletions in MIDV SA25/11 compared to other sequences are indicated by “^”.

Clinical signs for the six MIDV full genome strains are summarized in Table 5. A full genome sequence for ZRU080/14 and a near full genome sequence (missing the first 30 nucleotides of the 5′ UTR) was obtained for ZRU089/14 from blood samples without culturing. The other four full genome sequences were obtained through culturing on Vero cells for ZRU044/17, ZRU059/17/1, ZRU075/17, and ZRU103/17. P-distance analysis comparisons of the currently described MIDV strains to previous MIDV strains varied from 97.7–98.9% with all samples sharing the least similarity with the historic strain isolated from an *Ae. vittatus* collected in 1977 in Cairo (MIDV strain ArB-8422). Currently described MIDV strains showed the greatest percent amino identity with the strain from the Zimbabwean horse (MIDV 857; 98.7–98.9%) followed closely by the South African strain (MIDV SAE25/11; 98.2–98.9%) and even greater percentage identities between currently described strains (99.3–100%), especially from the same year (99.9–100%) (Supplementary Table S2). Samples from 2014, ZRU080/14 and ZRU089/14, shared 99.9% identity on nucleotide (nt) and amino acid (aa) level, while samples from 2017 (ZRU044/17, ZRU059/17/1, ZRU075/17, and ZRU103/17) all shared >99% identity on both nt and aa level. ZRU044/17 and ZRU059/17/1 were identical, while ZRU075/17 and ZRU103/17 shared 99.9% aa identity when comparing ORF’s. Both the non-structural and structural proteins shared high percentages of identity and overall, the nsP3 region showed the greatest divergence (98.49–99.35%) and the E3 the greatest similarity (100%) amongst MIDV strains. Phylogenetic analysis placed MIDV in its own complex, with strong posterior probability support for all phylogenetic trees (Figure 3, Figure 4, Figure 5, Figure 6 and Figure 7). Amino acid changes and positions comparing currently described MIDV strains and other published MIDV strains to that of MIDV SAE25/11 are indicated in Figure 8. Changes unique to the currently described six whole genomes were observed in the nsP3, nsP4, capsid, E2, E1, 5′UTR and 3′UTRregions.

Strains from 2017 share unique amino acid differences in the nsP3 (T59I, Q356L, R424G), nsP4 (V35A, T119I), capsid (A91T), 5′UTR (C33-) and 3′UTR (A80G, C114G) regions that are not seen in the two currently described strains from 2014 or historic MIDV strains. All sequences currently described contain the 3′UTR repeats that have been identified in MIDV strains isolated from horses but absent from historic arthropod strains [12,13,37].

### 3.6. Recombination Event Analysis

Using the RDP4 program, potential recombination events were detected in all six currently described full genome MIDV strains involving an event in the structural protein region (*p* < 0.05) at nucleotide position 2583–3092 (509 nt) of the structural protein (nucleotide position according to ZRU044/17). Semliki Forest virus (SFV) was identified as a potential major parent, while the minor parent remained unknown. The recombination event was detected by three out of the five (RDP, MaxChi, Chimaera, SciScan and 3Seq) detection models used during the current analysis within the RDP4 program (Table 6).

## 4. Discussion

MIDV and SINV have previously been reported in horses and wildlife from Africa with febrile and neurological diseases, as well as fatalities [11,12,13,14]. However, there are currently only four full genome sequences available for MIDV and the pathogenesis and clinical presentation of MIDV and SINV infections in animals is not well known. Although the amplification host for SINV is known to be birds, the reservoir and amplification hosts for MIDV are not yet defined. In addition, the period that SINV and MIDV replicate in horses, which contributes to their potential in reinfection of mosquitoes, is not known. The current study aimed to add to the limited epidemiological data available for these viruses and has resulted in six additional full/near full MIDV genomes.

Between January 2014–December 2018, 80 alphavirus positive cases (MIDV *n* = 69, SINV *n* = 11) were detected in horses from South Africa that presented with acute fever and/or neurological signs or fatalities related to these signs. Only whole blood samples were received for SINV positive specimens, but neurological manifestations were observed in 54.54% of SINV infections of which two had MIDV co-infections. The detection of MIDV in CNS tissue suggests it may have the ability to cross the blood-brain barrier which may play a role in neurological manifestations as observed with 73.91% of MIDV positives. MIDV was also detected in placenta and brain tissue of a foetus following abortion in a mare from the Gauteng province with no other signs specified for the mare. Although the fatality rate was lower for MIDV (8.70%) than has previously been reported for WNV (30.00%) [38], MIDV infection was positively associated with fatalities (*p* < 0.05), with 4/6 MIDV fatalities being MIDV/WNV co-infections indicating that MIDV co-infections could contribute to a more severe clinical outcome for other infections. MIDV has also been detected in humans that presented with neurological signs and whom had other co-morbidities at the time of testing [39]. Other signs that were positively associated with MIDV cases included: fever 75.36% (*p* < 0.001), icterus (28.99%; *p* < 0.001), anorexia/inappetence (28.99%; *p* < 0.05) and pallor (14.5%; *p* < 0.05).

The low number of SINV cases is likely due to the short period of viremia and low viral loads previously reported for SINV [40] and detection will most likely increase with implementation of serological techniques. No similar data is currently available for MIDV. Currently no IgM assays are available for SINV or MIDV in horses. The successful detection of MIDV via nested-PCR in samples taken up to 21 days after onset of signs suggests that MIDV has a long period of viral replication and that horses might, during this time, have the potential to act as amplification hosts for reinfection of mosquitoes.

An increase in the detection of alphaviruses in mosquito vectors and infections in humans and animals have previously been reported following heavy rainfalls [41,42,43,44]. South Africa has a summer rainfall pattern (November to April), with the exception of the Western Cape which has winter rainfalls and KwaZulu Natal and lowlands of Mpumalanga that have both summer and winter rainfalls. In general, the amount of precipitation increases from the west to the east [45]. MIDV infection was detected yearly from 2014–2018 with most MIDV positive cases detected in 2017 (9.84%, 42/427) followed by 2018 (5.15%; 7/136). MIDV was detected year-round with an increase in the number of cases seen in the late summer till early autumn months from February to May. The increase in MIDV infections during 2017 correspond with an increase in rainfall, including floods in numerous regions, from mid-2016 to February 2017 [46]. The increase in MIDV positive cases following heavy rains could be related to an increase in mosquito vectors since an increase in rainfall results in more mosquito eggs that hatch and develop into adult mosquitoes that act as vectors for viral transmission [47]. The highest MIDV positivity was seen in March (9.67%, 24/248) and April (7.38%; 13/176). MIDV infection was detected in all provinces except Limpopo. For SINV cases the majority were detected in 2015 (4.06%; 8/197) followed by 2014 (1.03%; 2/195) and 2018 (0.76%, 1/136) with no SINV cases detected during 2016 and 2017. SINV was detected year-round except in January, May, September, and November with the highest percent positivity seen in August (4.9%, 3/61). SINV positives were detected in only five of the nine South African provinces. Since only 11 SINV cases were detected between 2014–2018, no clear seasonality or geographic distribution could be determined or SINV.

Two of the 4 MIDV positive follow-up samples still presented with fever and icterus, respectively, 4 and 7 days after sampling of the initial sample. Analysis of the nsP4 and E1 amplicons showed no unique features for currently described MIDV strains that remained PCR positive for extended periods in comparison to other MIDV strains in the present study. It should be noted that follow-up specimens were requested for all MIDV positives during 2017/2018 but could only be obtained for 18/49 MIDV positives and 1/1 SINV positive detected in 2017/2018 as it was dependent on the veterinarian’s ability/willingness to collect a follow-up sample for each case. Thus, obtaining more follow-up samples for MIDV and SINV positive horses at set dates with detail of clinical signs during and following initial infection would provide more information surrounding the period of viremia for MIDV and SINV and the clinical relevance of this finding.

Phylogenetic analysis of the nsP4 fragment placed currently described SINV strains within the WEE complex. SINV strains described in the current study formed a distinct cluster distinguishing it from previously described SINV strains. An analysis of the E1 and nsP4 fragments placed MIDV within the MID complex. An analysis of the E1 fragments showed strains from the same year mostly clustering together, while that of the nsP4 fragment showed strains from the same year separated into different clusters and mixed with strains collected during other years. The E1 protein is essential for fusion of the viral envelope with the host endosomal membrane [48] and the importance of this protein has been demonstrated when a single E1-A226V mutation in CHIKV resulted in adaptation of CHIKV to the *Ae. albopictus* vector as opposed to the usual major mosquito vector, *Ae. aegypti*, which would aid in transmission of CHIKV due to the widespread distribution of *Ae. albopictus* [49]. MIDV has been detected in different mosquitoes across Africa [50], but the role of the E1 protein in MIDV vector adaptation has not been investigated.

Full genome sequence analysis of MIDV strains placed all currently described strains in the MID complex with strains of the same year again clustering together (Figure 5 and Figure 7). The two 2014 samples, that shared 99.9% aa identity, both presented with neurological signs (although different) while locations differed. Samples from 2017, ZRU 044/17 and ZRU059/17/1, shared 100% aa identity and shared location and clinical signs while ZRU075/17 and ZRU103/17 with 99.9% aa identity shared location but differed in clinical signs. ZRU075/17 had febrile and neurological manifestations while ZRU103/17 only presented with fever (Table 5). ZRU075/17 had the nsP2-S88G mutation, but there were no unique neurological signs in this horse when compared to the other strains where horses also presented with neurological signs without this mutation. Furthermore, the possibility that this mutation occurred due to adaptive mutation during culturing cannot be excluded, although none of the other cultures shared this mutation and were subjected to the same conditions. It should, however, be noted that the nsP2 alphavirus protein has been implicated as a virulence factor that can reduce the development of an antiviral response from the host [51,52]. The historic MIDV857 strain isolated from the horse that died following AHSV signs in Zimbabwe had 4 different mutations in the nsP2 protein not seen in currently described strains or historic arthropod vector strains. Mutations also occurred in the UTR that were different in ZRU075/17 when compared to the other strains (Figure 8). However, more data is needed to determine the significance of these mutations.

Recombination analysis using the RDP4 program identified possible recombination events within the E1 region with SFV as the potential “major parent”/sequence donor for all six currently described MIDV full genome strains. The E1 region has previously been detected as a possible recombination product of events between members of the SFV complex [12]. Unfortunately, the E1 amplicon obtained for *n* = 45 MIDV positives is too small to use for recombination analysis and thus only the 6 MIDV full genomes could be used for analysis.

Following an outbreak of SFV infection in the Central African Republic reported symptoms included fever, headache, myalgia and arthralgia [53]. A death following a laboratory infection with a virulent strain has also been reported [54]. Avirulent strains have been found to result in non-lethal demyelination in adult mice while these avirulent strains were still found to be abortogenic in pregnant mice [55]. Sporadic animal infections have yet to be reported for SFV.

MIDV and SINV have been detected in different wildlife, livestock, bird and equine species [13,14] with neurological disease and fatalities as supported by findings in the present study. Limitations of this study include the exclusion of all other possible infectious and non-infectious aetiologies in MIDV and SINV positive cases, use of semi-quantitative and qualitative rather than quantitative PCR and the specific selection for febrile and neurological cases. Recently, MIDV and SINV have been detected in the lungs of wildlife that died with pulmonary signs [14] and despite not selecting for these samples specifically MIDV and SINV infection was detected in horses that presented with respiratory signs including congested mucous membranes, tachypnoea and tachycardia for MIDV infection and nasal discharge for SINV infection. The historic MIDV857 case from the Zimbabwean horse had typical AHSV signs while suspected AHSV cases were not included in this investigation. Horses have been shown to have the capacity to transmit the new world alphavirus VEEV to mosquitoes and have a prolonged replication time, thus posing a greater risk for zoonotic infections to humans. Although further investigations are needed to confirm this is the case for MIDV, care should be taken to protect positive horses from mosquito bites during outbreaks and when handling blood from infected animals. This is contrary to WNV or SINV which have a short viremia in humans and horses and are both thought to be dead-end hosts [40,56]. The use of sentinel species, such as horses, is valuable in predicting large outbreaks and spill over events to humans. The large number of positive MIDV cases detected in horses presenting with acute neurological and febrile illness, especially during 2017, correlated with the detection of MIDV in humans during the same year, as recently published [39]. Development of serological analyses, especially for SINV which has a short period of viremia, would be very useful for obtaining more accurate estimates of prevalence by detecting past or recent infections.

## 5. Conclusions

Despite a lower fatality rate than viruses such as WNV, MIDV was associated with a high incidence of cases with neurological signs in horses and contributed significantly to morbidity in these animals in South Africa. MIDV has been isolated from mosquitoes across many parts of Africa [57]. No data currently exists on the importance of this virus in other parts of the continent. MIDV should also be monitored as a potential emerging disease in new regions. Reservoir host and vector competence will determine its potential to emerge in new regions. Although SINV was also detected in cases of neurological disease and death in horses, fewer cases were detected relative to MIDV by RT-PCR during the study period. An extended replication period for MIDV suggests that molecular diagnostic assays will detect more cases than is the case for SINV. The current study was not structured to provide information on the required level of viremia in horses to infect mosquitoes. However, the fact that MIDV could still be detected after an extended period in positive horses by RT-PCR in an area where a large number of MIDV cases were reported in 2017–2018 warrants further investigation as to their potential as amplifying hosts.

Detection of SINV infections was not as common but surveillance would benefit from addition of IgM diagnostic assays. Although more associated with fever, SINV also appear to be associated with neurological infections in horses while both viruses may also present with signs of arthralgia and respiratory signs in these animals.

## Figures and Tables

**Table 1:** Primers and probes used for detection of alphaviruses targeting a conserved region of the nsP4 gene in alphaviruses and the E1 region of MIDV specifically. Nucleotide positions are indicated for MIDV (Genbank accession number KF680222.1) and SINV (Genbank accession number U38305) genomes.

PCR	Primer Name (Orientation/Channel Dye)	Sequence 5′–3′	Position (MIDV/SINV)	Region and Amplicon Size	Reference
Alpha first round	Alpha1+ (sense)	GAYGCITAYYTIGAYATGGTIGAIGG	5888–6368/ 6162–6642	nsP4 480 bp	Sánchez-Seco et al., 2001 [15]
	Alpha1− (antisense)	KYTCYTCIGTRTGYTTIGTICCIGG			
Alpha nested	Alpha2+ (sense)	GIAAYTGYAAYGTIACICARATG	6066–6264/ 6340–6538	nsP4 198 bp	
	Alpha2− (antisense)	GCRAAIARIGCIGCIGCYYTIGGICC			
	MIDV probe	GCTTTAAGAAGTACGCATGCAACA	6132–6155	nsP4 N/A	van Niekerk et al., 2015 [13]
	SINV probe	ATGACGAGTATTGGGAGGAGTTTG	6427–6450		
Nested specific MIDV	MNF (sense	GCAGCCTTTTGTCCGTCYAA	5936–6283	nsP4 347 bp	Steyn et al., 2020 [14]
	MNR (antisense)	GGCTTCAAGTCRTAGGTTT			
Nested specific SINV	SNF (sense)	GCAACCTTYTGCCCCGCYAA	6209–6556	nsP4 347 bp	
	SNR (antisense)	GGGACCAAATTATRCGTCT			
MIDV E first round	MIDV EF (sense)	TTGTCAACGGAGAGAGCAC	10,231–11,048	E1 817 bp	This study
	MIDV ER (antisense)	CTATGGGCGGAGCTACTGTG			
MIDV E nested	MIDV EN 9F (sense)	ACCGGGTAGATTTGGGGACT	10,379–10,930	E1 551 bp	This study
	MIDV 10,911 EN (antisense)	CACTTTGCTGTGCAAGTGGT			van Niekerk et al., 2015 [13]

MIDV: Middelburg virus; SINV: Sindbis virus.

**Table 2:** Summary of MIDV and SINV detected in fatal cases in horses from South Africa between January 2014–December 2018.

MIDV and SINV Positive Fatal Cases in Horses January 2014–December 2018							
	Year	2014	2015	2016	2017	2018	Total
Virus	No. samples received	195	197	129	427	136	1084
MIDV	% Positive	4.61% (9/195)	5.07% (10/197)	077% (1/129)	9.84% (42/427)	6.48% (7/108)	6.36% (69/1084)
	% Fatal	22.22% (2/9)	10.00% (1/10)	0.00% (0/1)	7.14% (3/42)	0.00% (0/7)	8.69% (6/69)
	% Fatal co-infections	100% (2 WNV/2)	0.00% (0/1)	N/A	66.66% (2 WNV/3)	N/A	66.66% (4/6)
SINV	% Positive	1.02% (2/195)	4.06% (8/197)	0.00% (0/0) (0/129)	0.00% (0/427)	0.92% (1/108)	1.01% (11/1084)
	% Fatal	100% (2/2)	0.00% (0/8)	0.00% (0/0)	0.00% (0/0)	0.00% (0/1)	18.18% (2/11)
	% Fatal co-infections	50.00% (1 AHSV/EEV/2)	0.00% (0/0)	0.00% (0/0)	0.00% (0/0)	0.00% (0/0)	50.00% (1/2)

MIDV: Middelburg virus; SINV: Sindbis virus; AHSV: African horse sickness virus; EEV: Equine encephalosis virus, WNV: West Nile virus; N/A: Not applicable.

**Table 3:** Main clinical signs observed in MIDV and SINV positive horses from South Africa between January 2014–December 2018.

Middelburg	Sign	MIDV Positive *n* = 69(%)	MIDV Negative *n* = 1015 (%)	*p*-Value	Odds Ratio (95% CI)
	**Died or euthanized**	**6 (8.70)**	**235 (23.15)**	**0.004**	0.31 (0.13–0.73)
Neurological signs	Any neurological signs	51 (73.91)	667 (65.71)	0.188	1.48 (0.85–2.59)
	Ataxia	34 (49.23)	388 (38.23)	0.074	1.56 (0.96–2.55)
	Paresis	11 (15.94)	122 (12.02)	0.342	1.38 (0.70–2.71)
	Hind leg paralysis	6 (8.70)	47 (4.63)	0.142	1.96 (0.80–4.76)
	Recumbency	6 (8.70)	159 (15.67)	0.163	0.51 (0.21–1.20)
	Paralysis	3 (4.35)	97 (9.56)	0.195	0.43 (0.13–1.39)
	Tremors/fasciculations	3 (4.35)	28 (2.76)	0.441	1.6 (0.47–5.40)
	Seizures	1 (1.45)	49 (4.83)	0.365	0.28 (0.03–2.13)
	**Stiffness**	**6 (8.70)**	**7 (0.69)**	**0.0001**	13.71 (4.47–42.02)
Fever	**Fever**	**52 (75.36)**	**529 (52.12)**	**0.00025**	**2.81 (1.60–4.92)**
	Fever only	13 (18.84)	168 (16.55)	1.000	1.015 (0.543–1.895)
	**Fever and neurological signs**	**38 (55.07)**	**188 (18.52)**	**2 × 10^−10^**	**5.25 (3.18–8.66)**
	Fever, neurological and respiratory signs	1 (1.45)	3 (0.29)	0.235	4.85 (0.49–47.38)
Other	**Icterus**	**20 (28.99)**	**118 (11.63)**	**0.00021**	**3.1 (1.78–5.40)**
	**Anorexia/inappetence**	**20 (28.99)**	**189 (18.62)**	**0.039**	**1.78 (1.03–3.07)**
	**Pallor**	**10 (14.49)**	**69 (5.91)**	**0.010**	**2.69 (1.31–5.53)**
**Sindbis**	**Sign**	**SINV Positive *n* = 11 (%)**	**SINV Negative *n*= 1073 (%)**	***p*-Value**	**Odds Ratio (95% CI)**
	Died or euthanized	2 (18.18)	239 (22.27)	1	0.77 (0.16–3.61)
Neurological signs	Any neurological signs	6 (54.54)	713 (66.45)	0.522	0.42 (0.12–1.38)
	Ataxia	3 (27.27)	419 (39.05)	0.543	0.58 (0.15–2.21)
	Recumbency	2 (18.18)	163 (15.19)	0.678	1.24 (0.26–5.79)
	Circling/paddling	1 (9.09)	17 (1.58)	0.168	6.21 (0.75–51.27)
	**Facial nerve paralysis**	**1 (9.09)**	**3 (0.28)**	**0.040**	**35.67 (3.41–372.98)**
	Seizures	1 (9.19)	49 (4.57)	0.406	2.08 (0.26–16.65)
	Tongue paralysis	1 (9.09)	7 (0.65)	0.078	15.22 (1.71–135.51)
	Tremors/fasciculations	1 (9.09)	30 (2.80)	0.274	3.47 (0.43–28.03)
Fever	Fever	8 (72.73)	573 (53.40)	0.237	2.33 (0.61–8.81)
	Fever only	3 (27.27)	179 (16.68)	0.406	1.88 (0.49–7.17)
	Fever and neurological signs	4 (36.36)	220 (20.50)	0.260	2.13 (0.61–7.36)
	Fever, neurological and respiratory signs	0 (0.00)	4 (0.37)	1.000	Undefined(undefined)
Other	Anorexia/inappetence	4 (36.36)	205 (19.11)	0.237	2.41 (0.71–8.34)
	Flaccid tail	1 (9.09)	7 (0.65)	0.078	15.22 (1.71–135.51)
	Icterus	1 (9.09)	137 (12.77)	1	0.68 (0.08–5.37)
	**Nasal discharge**	**2 (18.18)**	**19 (1.78)**	**0.021**	**12.26 (2.48–60.64)**

MIDV: Middelburg virus; SINV: Sindbis virus; CI: Confidence Interval. Signs significantly associated with viral infection are indicated in bold.

**Table 4:** Detection of MIDV in original and follow-up specimens from horses from South Africa between January 2014–December 2018 via real-time RT-PCR.

Sample Group.	Horse 1	Horse 2	Horse 3	Horse 4
Sample ID’s	ZRU074/17 ZRU141/17/6	ZRU060/17 ZRU141/17/1	ZRU034/18 ZRU031/18 ZRU038/18	ZRU194/17/3 ZRU209/17
Date sick	2017/03/07	2017/03/03	2018/02/03	2017/04/03
Date first sampled	2017/03/07	2017/03/03	2018/02/05	2017/04/03
Days from onset for first sample (PCR positive)	0 days	0 days	2 days	0 days
Initial signs	Fever & Icterus	Ataxia, Fever & Icterus	Fever	Anorexia/inappetence, Fever, Icterus, Pallor
Virus result on first submission (test method)	MIDV/EEV (PCR)	MIDV (PCR)	MIDV (PCR)	MIDV (PCR)
Date follow-up sampled	2017/03/24	2017/03/24	2018/02/07 2018/02/17	2017/04/10
Days from onset for follow-up sample (PCR positive)	17 days	21 days	4 and 14 days	7 days
Signs at follow-up	Healthy	Healthy	Fever, Healthy	Icterus
Virus result for follow-up specimen (test method)	MIDV/EEV (PCR)	MIDV (PCR)	MIDV (PCR)	MIDV/WNV (MIDV PCR/WNV IgM ELISA)
Location	Benoni	Benoni	Bapsfontein	Onderstepoort

EEV: Equine encephalosis virus; MIDV: Middelburg virus; WNV: West Nile virus.

**Table 5:** Clinical signs for MIDV positive horses for which full genomes were obtained.

ZRU Number	Year	Signs	Location (Province, City)	Co-Infection	Clinical Outcome
ZRU080/14	2014	Stiffness	North West, Vryburg	None detected	Alive
ZRU089/14	2014	Ataxia, icterus, paresis, fever	Free State, Ladybrand	AHSV	Alive
ZRU044/17	2017	Ataxia, icterus, fever	Gauteng, Pretoria	None detected	Alive
ZRU059/17/1	2017	Ataxia, icterus, fever	Gauteng, Pretoria	None detected	Alive
ZRU075/17	2017	Ataxia, fever	Gauteng, Benoni	None detected	Alive
ZRU103/17	2017	Fever	Gauteng, Benoni	None detected	Alive

AHSV: African horse sickness virus; MIDV: Middelburg virus

**Table 6:** Possible recombination events associated with currently described MIDV full genomes (ZRU080/14; ZRU089/14; ZRU044/17; ZRU059/17/1; ZRU075/17 and ZRU103/17) within the structural region. Only detection models with significant statistical support (*p* < 0.05) are shown. Nucleotide positions corresponds to the structural proteins (C+E3+E2+6k+E1 excluding the 5′UTR and 3′UTR) of ZRU044/17. SFV Semliki Forest Virus (Genbank accession number CAA27742); nt = nucleotide.

Possible Event	Parental Sequence		RDP4 Recombination Detection Model and Corresponding *p*-Values				
SP nt position	Major	Minor	RDP	MaxChi	Chimaera	SciScan	3Seq
2582–3093 (509 nt)	SFV	Unknown	1.833 × 10^−2^	3.921 × 10^−4^	8.034 × 10^−4^	5.921 × 10^−11^	1.242 × 10^−9^

## Data Availability

All sequence data can be obtained from https://www.ncbi.nlm.nih.gov/genbank using the Genbank accession numbers indicated in Supplementary Table S1.

## Supplementary Materials

The following supporting information can be downloaded at: https://www.mdpi.com/article/10.3390/v14092013/s1, Table S1: GenBank accession numbers for members of the Alphavirus genus used for phylogenetic analysis in this study. Sequences obtained in the current study are in bold font.; Table S2: Comparison of percentage identity of individual proteins and Middelburg virus full genome isolated from horses in the present study to previously identified Middelburg virus strains. Nucleotide and amino acid identities are shown for complete (concatenated) genomes with amino acids in the lower left matrix and nucleotides in the upper right matrix. Only amino acid identities are shown for individual proteins.
