# Supplementary material for: Epidemiological and Genomic Characterisation of Middelburg and Sindbis Alphaviruses Identified in Horses with Febrile and Neurological Infections, South Africa (2014–2018)
Source: Viruses. 2022 Sep 11;14(9):2013. doi: 10.3390/v14092013 (PMC9501102; doi:10.3390/v14092013)
Supplement: Supplementary file 1 [file viruses-14-02013-s001.zip › viruses-1852770-supplementary.pdf]

Table S1. GenBank accession numbers for members of the *Alphavirus* genus used for phylogenetic analysis in this study. Sequences obtained in the current study are in bold font.

Antigenic complex	Species	Host	Location	GenBank accession number (strain name and gene where applicable)
		Reference strains		
Middelburg	Middelburg virus	Horse	South Africa	KF680222.1 (SAE25/11)
		Horse	Zimbabwe	EF536323.1 (857)
		Ae. vittatus	CAR	KM115530.1 (ArB-8442)
		Am. variegatum	CAR	KM115531.1 (ArTB-5290)
Western equine encephalitis	Aura virus	*Culex (melanoconion) spp*	South America	AF126284
	Sindbis virus	*Culex annulirostris*	Australia	AF492770
		Arthropod	South Africa	U38305
	Trocara virus	*Culex serratus*	Brazil	HM147991
	Whataroa virus	*Cx. Spp*	New Zealand	HM147993.1
Eastern equine encephalitis	EEEV Lin I NA	Mosquito	USA	EF151502.1
	EEEV Lin II SA	Mosquito	Peru	DQ241303.1
	EEEV Lin III SA	Mosquito	Peru	DQ241304.1
Venezuelan Equine Encephalitis	Venezuelan Equine Encephalitis virus	Mosquito	Venezuela	L04653.1
		Mosquito	Venezuela	L04653
	Everglades	*Cx melanoconion sp*	USA	AF075251.1
	Mucambo virus		Brazil	AF075253.1
	Tonate virus		Guyana	AF075254.1
Barmah Forest	Barmah Forest virus	*Cx. annulirostris*	Australia	MK697274.1
Ndumu	Ndumu virus	*Ae. Spp*	Africa	NC_016959.1
Semliki Forest	Semliki Forest virus	*Ae. abnormalis group*	Africa	X04129.1
	Chikungunya virus	Human	Africa	AF369024.2
	O'nyong-nyong virus	Human	Uganda	AF079456.1
	Mayaro virus	Human	French Guiana	KJ013266.2
	Ross river virus	*Culex and Aedes spp*	Australasia	GQ433358
	Sagiyama virus	*Culex and Aedes spp*	Japan	AB032553
	Getha virus	*Culex and Aedes spp*	Australasia	AY702913
Taï Forest alphavirus	Taï Forest alphavirus			NC_032681
Eilat	Eilat virus	*An. coustani*	Israel	NC_018615.1
Sleeping disease	Sleeping disease virus	Rainbow trout	France	AJ316246
Southern elephant seal	Southern elephant seal virus	Seal	Australia	NC_016960.1
		**Current study**		
Middelburg	Middelburg virus	**Horse**	South Africa	**MT015693 (ZRU080/14 full genome)**
				**MT015692 (ZRU089/14 full genome)**
				**MT015691 (ZRU044/17 full genome)**
				**MT015690 (ZRU059/17/1 full genome)**
				**MT015689 (ZRU075/17 full genome)**
				**MT015688 (ZRU103/17 full genome)**
				**MT041775 (ZRU098/17 nsP4)**
				**MT041776 (ZRU116/14/1 nsP4)**
				**MT041777 (ZRU090/18/2 nsP4)**

	MT041778 (ZRU089/18 nsP4)
	MT041779 (ZRU090/18/5 nsP4)
	MT041780 (ZRU122/18 nsP4)
	MT041781 (ZRU055/17 nsP4)
	MT041782 (ZRU059/17/2 nsP4)
	MT041783 (ZRU074/17 nsP4)
	MT041784 (ZRU076/17 nsP4)
	MT041786 (ZRU129/17 nsP4)
	MT041787 (ZRU240/17 nsP4)
	MT041788 (ZRU107/17/3 nsP4)
	MT041789 (ZRU140/17 nsP4)
	MT041790 (ZRU114/17 nsP4)
	MT041791 (ZRU158/17 nsP4)
	MT041792 (ZRU495/17 nsP4)
	MT041793 (ZRU041/17 nsP4)
	MT041794 (ZRU141/17/6 nsP4)
	MT041795 (ZRU209/17 nsP4)
	MT041796 (ZRU211/17 nsP4)
	MT041797 (ZRU070/17 nsP4)
	MT041798 (ZRU084/17 nsP4)
	MT041799 (ZRU348/17 nsP4)
	MT041800 (ZRU107/17/4 nsP4)
	MT041801 (ZRU069/17 nsP4)
	MT041802 (ZRU078/17 nsP4)
	MT041803 (ZRU053/17 nsP4)
	MT041804 (ZRU194/17/3 nsP4)
	MT041805 (ZRU141/17/1 nsP4)
	MT041806 (ZRU019/17 nsP4)
	MT041807 (ZRU205/17 nsP4)
	MT041808 (ZRU298/17 nsP4)
	MT041809 (ZRU325/17 nsP4)
	MT041810 (ZRU109/14 nsP4)
	MT041811 (ZRU110/14 nsP4)
	MT041812 (ZRU129/14/5 nsP4)
	MT041813 (ZRU213/15 nsP4)
	MT041814 (ZRU052/17 nsP4)
	MT041815 (ZRU247/17 nsP4)
	MT041816 (ZRU180/14/2 nsP4)
	MT041817 (ZRU089/15 nsP4)
	MT041818 (ZRU244/17 nsP4)
	MT041819 (ZRU067/17 nsP4)
	MT041820 (ZRU037/17/2 nsP4)
	MT041821 (ZRU034/17/1 nsP4)
	MT041822 (ZRU060/17 nsP4)
	MT041823 (ZRU238/17 nsP4)
	MT041824 (ZRU110/18 nsP4)
	MT041825 (ZRU053/18 nsP4)
	MT041826 (ZRU207/15 nsP4)
	MT041827 (ZRU034/18 nsP4)
	MT041828 (ZRU029/18 nsP4)
	MT068150 (ZRU014/15 E1)

				**MT068151 (ZRU053/18 E1)**
				**MT068152 (ZRU029/18 E1)**
				**MT068153 (ZRU122/18 E1)**
				**MT068154 (ZRU089/18 E1)**
				**MT068155 (ZRU034/18 E1)**
				**MT068156 (ZRU089/15 E1)**
				**MT068157 (ZRU495/17 E1)**
				**MT068158 (ZRU034/17/1 E1)**
				**MT068159 (ZRU034/17/2 E1)**
				**MT068160 (ZRU067/17 E1)**
				**MT068161 (ZRU019/17 E1)**
				**MT068162 (ZRU037/17/2 E1)**
				**MT068163 (ZRU041/17 E1)**
				**MT068164 (ZRU060/17 E1)**
				**MT068165 (ZRU069/17 E1)**
				**MT068166 (ZRU078/17 E1)**
				**MT068167 (ZRU084/17 E1)**
				**MT068168 (ZRU140/17 E1)**
				**MT068169 (ZRU158/17 E1)**
				**MT068170 (ZRU209/17 E1)**
				**MT068171 (ZRU240/17 E1)**
				**MT068172 (ZRU247/17 E1)**
				**MT068173 (ZRU194/17/3 E1)**
				**MT068174 (ZRU059/17/2 E1)**
				**MT068175 (ZRU107/17/3 E1)**
				**MT068176 (ZRU052/17 E1)**
				**MT068177 (ZRU053/17 E1)**
				**MT068178 (ZRU055/17 E1)**
				**MT068179 (ZRU107/17/4 E1)**
				**MT068180 (ZRU114/17 E1)**
				**MT068181 (ZRU325/17 E1)**
				**MT068182 (ZRU348/17 E1)**
				**MT068183 (ZRU070/17 E1)**
				**MT068184 (ZRU129/14/5 E1)**
				**MT068185 (ZRU110/14 E1)**
				**MT068186 (ZRU180/14/2 E1)**
				**MT068187 (ZRU109/14 E1)**
				**MT068188 (ZRU244/17 E1)**
Western equine encephalitis	Sindbis virus	**Horse**	South Africa	**MT068189 (ZRU026/15 nsP4)**
				**MT068190 (ZRU104/15 nsP4)**
				**MT068191 (ZRU113/14 nsP4)**
				**MT068192 (ZRU136/15/9 nsP4)**
				**MT068193 (ZRU247/14 nsP4)**
				**MT068194 (ZRU165/15/8 nsP4)**
				**MT068195 (ZRU030/18 nsP4)**

Cx. = Culex; Ae. = Aedes; An. = Anopheles, nsP = non-structural Protein

Table S2. Comparison of percentage identity of individual proteins and Middelburg virus full genome isolated from horses in the present study to previously identified Middelburg virus strains. Nucleotide and amino acid identities are shown for complete (concatenated) genomes with amino acids in the lower left matrix and nucleotides in the upper right matrix. Only amino acid identities are shown for individual proteins.

MIDV Strain	SAE 25/11	857	ArB-8422	ArTB-5290	ZRU080/14	ZRU089/14	ZRU044/17	ZRU059/17/1	ZRU075/17	ZRU103/17
Concatenated ORF1 and 2										
SAE25/11		98.63%	97.75%	98.62%	98.90%	98.83%	98.26%	98.26%	98.27%	98.28%
857	98.63%		98.33%	99.03%	98.89%	98.86%	98.72%	98.72%	98.73%	98.75%
ArB-8422	97.71%	98.30%		98.37%	97.92%	97.90%	97.72%	97.72%	97.73%	97.75%
ArTB-5290	98.58%	99.01%	98.30%		98.77%	98.72%	98.57%	98.57%	98.57%	98.59%
ZRU080/14	98.88%	98.91%	97.91%	98.78%		99.91%	99.34%	99.34%	99.35%	99.36%
ZRU089/14	98.80%	98.87%	97.88%	98.71%	99.91%		99.36%	99.36%	99.35%	99.36%
ZRU044/17	98.24%	98.71%	97.69%	98.55%	99.34%	99.36%		100%	99.94%	99.95%
ZRU059/17/1	98.24%	98.71%	97.69%	98.55%	99.34%	99.36%	100%		99.94%	99.95%
ZRU075/17	98.24%	98.71%	97.69%	98.55%	99.34%	99.36%	99.91%	99.91%		99.98%
ZRU103/17	98.27%	98.75%	97.72%	98.58%	99.37%	99.38%	99.95%	99.95%	99.97%	
nsP1										
SAE25/11										
857	99.26%									
ArB-8422	98.88%	98.88%								
ArTB-5290	99.07%	99.07%	98.70%							
ZRU080/14	99.63%	99.63%	99.26%	99.44%						
ZRU089/14	99.63%	99.63%	99.26%	99.44%	100%					
ZRU044/17	99.63%	99.63%	99.26%	99.44%	100%	100%				
ZRU059/17/1	99.63%	99.63%	99.26%	99.44%	100%	100%	100%			
ZRU075/17	99.63%	99.63%	99.26%	99.44%	100%	100%	100%	100%		
ZRU103/17	99.63%	99.63%	99.26%	99.44%	100%	100%	100%	100%	100%	
nsP2										
SAE25/11										
857	99.37%									
ArB-8422	99.62%	99.25%								
ArTB-5290	99.75%	99.37%	99.62%							
ZRU080/14	100%	99.37%	99.62%	99.75%						
ZRU089/14	100%	99.37%	99.62%	99.75%	100%					
ZRU044/17	99.87%	99.50%	99.75%	99.87%	99.87%	99.87%				
ZRU059/17/1	99.87%	99.50%	99.75%	99.87%	99.87%	99.87%	100%			
ZRU075/17	99.75%	99.37%	99.62%	99.75%	99.75%	99.75%	99.87%	99.87%		
ZRU103/17	99.87%	99.50%	99.75%	99.87%	99.87%	99.87%	100%	100%	99.87%	
nsP3										
SAE25/11										
857	99.14%									
ArB-8422	98.92%	99.35%								
ArTB-5290	99.35%	99.78%	99.57%							
ZRU080/14	99.35%	99.35%	99.14%	99.57%						
ZRU089/14	99.35%	99.35%	99.14%	99.57%	100%					
ZRU044/17	98.49%	98.92%	98.71%	99.14%	99.14%	99.14%				
ZRU059/17/1	98.49%	98.92%	98.71%	99.14%	99.14%	99.14%	100%			
ZRU075/17	98.49%	98.92%	98.71%	99.14%	99.14%	99.14%	100%	100%		
ZRU103/17	98.49%	98.92%	98.71%	99.14%	99.14%	99.14%	100%	100%	100%	
nsP4										
SAE25/11										
857	99.51%									
ArB-8422	99.67%	99.84%								
ArTB-5290	99.67%	99.84%	100%							
ZRU080/14	99.67%	99.84%	100%	100%						
ZRU089/14	99.67%	99.84%	100%	100%	100%					
ZRU044/17	99.34%	99.51%	99.67%	99.67%	99.67%	99.67%				
ZRU059/17/1	99.34%	99.51%	99.67%	99.67%	99.67%	99.67%	100%			
ZRU075/17	99.34%	99.51%	99.67%	99.67%	99.67%	99.67%	100%	100%		

ZRU103/17	99.34%	99.51%	99.67%	99.67%	99.67%	99.67%	100%	100%	100%
capsid									
SAE25/11									
857	98.89%								
ArB-8422	99.26%	99.63%							
ArTB-5290	99.26%	99.63%	100%						
ZRU080/14	99.26%	98.89%	99.26%	99.26%					
ZRU089/14	99.26%	98.89%	99.26%	99.26%	100%				
ZRU044/17	98.52%	98.89%	99.26%	99.26%	99.26%	99.26%			
ZRU059/17/1	98.52%	98.89%	99.26%	99.26%	99.26%	99.26%	100%		
ZRU075/17	98.52%	98.89%	99.26%	99.26%	99.26%	99.26%	100%	100%	
ZRU103/17	98.52%	98.89%	99.26%	99.26%	99.26%	99.26%	100%	100%	100%
E3									
SAE25/11									
857	100%								
ArB-8422	100%	100%							
ArTB-5290	100%	100%	100%						
ZRU080/14	100%	100%	100%	100%					
ZRU089/14	100%	100%	100%	100%	100%				
ZRU044/17	100%	100%	100%	100%	100%	100%			
ZRU059/17/1	100%	100%	100%	100%	100%	100%	100%		
ZRU075/17	100%	100%	100%	100%	100%	100%	100%	100%	
ZRU103/17	100%	100%	100%	100%	100%	100%	100%	100%	100%
E2									
SAE25/11									
857	99.29%								
ArB-8422	99.05%	99.76%							
ArTB-5290	99.05%	99.76%	100%						
ZRU080/14	99.05%	99.76%	99.52%	99.52%					
ZRU089/14	99.05%	99.76%	99.52%	99.52%	100%				
ZRU044/17	99.05%	99.76%	99.52%	99.52%	100%	100%			
ZRU059/17/1	99.05%	99.76%	99.52%	99.52%	100%	100%	100%		
ZRU075/17	99.05%	99.76%	99.52%	99.52%	100%	100%	100%	100%	
ZRU103/17	99.05%	99.76%	99.52%	99.52%	100%	100%	100%	100%	100%
6K									
SAE25/11									
857	100%								
ArB-8422	100%	100%							
ArTB-5290	98.39%	98.39%	98.39%						
ZRU080/14	98.39%	98.39%	98.39%	100%					
ZRU089/14	98.39%	98.39%	98.39%	100%	100%				
ZRU044/17	100%	100%	100%	98.39%	98.39%	98.39%			
ZRU059/17/1	100%	100%	100%	98.39%	98.39%	98.39%	100%		
ZRU075/17	100%	100%	100%	98.39%	98.39%	98.39%	100%	100%	
ZRU103/17	100%	100%	100%	98.39%	98.39%	98.39%	100%	100%	100%
E1									
SAE25/11									
857	99.77%								
ArB-8422	99.54%	99.77%							
ArTB-5290	99.08%	99.31%	99.08%						
ZRU080/14	99.54%	99.77%	99.54%	99.08%					
ZRU089/14	99.54%	99.77%	99.54%	99.08%	100%				
ZRU044/17	99.54%	99.77%	99.54%	99.08%	100%	100%			
ZRU059/17/1	99.54%	99.77%	99.54%	99.08%	100%	100%	100%		
ZRU075/17	99.54%	99.77%	99.54%	99.08%	100%	100%	100%	100%	
ZRU103/17	99.54%	99.77%	99.54%	99.08%	100%	100%	100%	100%	100%

MIDV = Middelburg virus; ORF = open reading frame; nsP = non-structural Protein.
